# Short Sleep Duration Is Associated with Shorter Telomere Length in Healthy Men: Findings from the Whitehall II Cohort Study

**DOI:** 10.1371/journal.pone.0047292

**Published:** 2012-10-29

**Authors:** Marta Jackowska, Mark Hamer, Livia A. Carvalho, Jorge D. Erusalimsky, Lee Butcher, Andrew Steptoe

**Affiliations:** 1 Research Department of Epidemiology and Public Health, University College London, London, United Kingdom; 2 Cardiff School of Health Sciences, Cardiff Metropolitan University, Cardiff, United Kingdom; Innsbruck Medical University, Austria

## Abstract

**Background:**

Shorter telomere length and poor sleep are more prevalent at older ages, but their relationship is uncertain. This study explored associations between sleep duration and telomere length in a sample of healthy middle and early old age people.

**Methods:**

Participants were 434 men and women aged 63.3 years on average drawn from the Whitehall II cohort study. Sleep duration was measured by self-report.

**Results:**

There was a linear association between sleep duration and leukocyte telomere length in men but not in women (P = 0.035). Men reporting shorter sleep duration had shorter telomeres, independently of age, body mass index, smoking, educational attainment, current employment, cynical hostility scores and depressive symptoms. Telomeres were on average 6% shorter in men sleeping 5 hours or fewer compared with those sleeping more than 7 hours per night.

**Conclusion:**

This study adds to the growing literature relating sleep duration with biomarkers of aging, and suggests that shortening of telomeres might reflect mechanisms through which short sleep contributes to pathological conditions in older men.

## Introduction

Mean telomere length is an indicator of biological age, and is modified by genetic as well as environmental factors [1], [2]. Shortening of telomeres has been linked with cardiovascular outcomes [3] as well as with inflammation [3], [4], low socioeconomic status [5], chronic stress [6], depression [7], hostility [8], smoking [9], [10] and obesity [10], [11].

Short sleep duration is also associated with cardiovascular disease [12], [13] and other health outcomes such as obesity [14], raised levels of inflammatory markers [15], and depressive symptoms [16]. To date the association between sleep duration and telomere length has only been studied in women [17]–[19], or in the context of sleep apnea [20], [21]. Therefore we explored associations between telomere length and self-reported sleep duration in a sample of healthy middle and early old age healthy men and women. Although men have shorter telomere length [4], [9] and experience poorer sleep than women [22], [23], the relationship between sleep duration and telomere length remains uncertain in men. Therefore this study took an explorative approach to test whether associations between sleep duration and telomere length would differ between men and women.

## Methods

### Study population

Participants were drawn from the Whitehall II epidemiological study [24] and were eligible if they were free from coronary heart disease, stroke, hypertension, inflammatory diseases, diabetes, active cancer and allergies. Disease status was ascertained through a telephone screening interview as well as by inspecting clinical data obtained in the previous 7 phases of the Whitehall II study. Data were collected between 2006 and 2008. All participants gave written consent, and the study was approved by the University College London Hospital committee on the Ethics of Human Research.

### Measures

Leukocyte relative mean telomere length was measured in triplicate by a monochrome multiplex Quantitative Real-Time PCR (Q-PCR) assay using a Bio-Rad CFX96™ Real-Time PCR Detection System (Bio-Rad, Hemel Hempstead, UK) (see [25] for more details).

Sleep duration was measured by asking participants to describe how many hours of sleep they have on an average weeknight, and responses were categorized into ‘5 hours or less’, ‘6 hours or less’, ‘7 hours or less’, ‘more than 7 hours’.

Depressive symptoms were measured with the Centre for Epidemiologic Studies Depression scale (CES-D) [26] and cynical hostility was assessed with the Cook Medley Hostility Scale [27]. Information about age, smoking, education, body mass index (BMI; kg/m^2^) and current employment status was also collected.

### Statistical analysis

Associations between telomere length and sleep duration were assessed with analysis of variance testing for linear contrasts. Since we have previously shown that shorter telomere length is associated with lower education [25] and greater hostility [8], these were included as covariates in addition to age, BMI, smoking, current employment status and depressive symptoms. Analyses were stratified by gender. Data were analyzed using SPSS version 18 (Chicago, Ill, USA).

## Results

There were 228 women and 206 men in the sample, and 31% of participants had a university degree (see Table 1). Around 6% of respondents smoked and BMI was 25.9 on average. 6.7% percent of the sample reported sleeping 5 or fewer hours on average, while 34.8% slept between 5 and 6 hours; 43.1% reported sleeping between 6 and 7 hours and the remainder slept longer than 7 hours (see Table 1).

**Table 1:** Characteristics of study participants.

Variable	Mean (SD)^a^/frequency (%)
Gender	
Male	206 (47.5%)
Female	228 (52.5%)
Age	63.3 (5.6)
Education attainment	
No qualification	34 (7.8%)
0-levels	113 (26.0%)
A/S levels	121 (27.9%)
Degree and above	135 (31.1%)
Paid work	
Yes	152 (35.0%)
No	282 (65.0%)
Current smoker	
Yes	27 (6.2%)
No	407 (93.8%)
BMI^b^ (kg/m^2^)	25.9 (4.0)
Cynical hostility (range 0–10)	2.6 (2.4)
Depressive symptoms (CES-D^c^; range 0–36)	6.7 (6.6)
Sleep duration	
≤5 hours	29 (6.7%)
≤6 hours	151 (34.8%)
≤7 hours	187 (43.1%)
>7 hours	67 (15.4%)

a SD = standard deviation.

b BMI = Body mass index.

c CES-D = Centre for Epidemiological Studies Depression scale.

Short sleep hours were more likely to be reported by men with the lowest level of education (P = 0.025), while women who were in paid employment were least likely to sleep longer than 7 hours (P = 0.010) (see Table 2). In both men and women short sleep duration was associated with more depressive symptoms (P = 0.031 and P<0.001, respectively), and with higher hostility scores in women (P = 0.034). Sleep duration was unrelated to gender in this sample (P = 0.302).

**Table 2:** Sleep duration and telomere length in men and women from the Whitehall II cohort study.

Variable	≤5 hours	≤6 hours	≤7 hours	>7 hours	P for linear trend
**Men (n = 206)** a					
Age (years)	63.8 (5.9)	61.6 (5.5)	62.9 (5.7)	63.6 (6.1)	0.898
BMI (kg/m^2^)	25.9 (2.2)	26.4 (3.7)	26.6 (4.1)	26.4 (3.2)	0.692
Current smoker (%)	0 (0)	8 (10.0)	3 (3.7)	2 (6.1)	0.310
Lowest level of education^b^ (%)	3 (27.3)	5 (6.8)	2 (2.5)	0 (0.0)	0.025
Paid employment (%)	5 (41.7)	42 (52.5)	33 (40.7)	9 (27.3)	0.092
Cynical hostility (range 0–10)	3.8 (2.2)	3.1 (2.5)	2.7 (2.6)	3.1 (2.8)	0.345
Depressive symptoms range (0–36)	8.5 (6.2)	7.8 (7.2)	5.2 (5.4)	4.8 (5.0)	0.031
Telomere length	0.956 (0.089)	0.986 (0.080)	0.990 (0.080)	0.999 (0.070)	0.099
Telomere length+all covariates^c^	0.948 (0.085)	0.988 (0.081)	0.990 (0.081)	1.008 (0.070)	0.035
**Women (n = 228)** a					
Age (years)	65.5 (5.3)	63.8 (5.8)	63.7 (5.2)	64.3 (5.4)	0.452
BMI (kg/m^2^)	24.9 (3.6)	25.1 (3.7)	25.6 (4.7)	25.2 (4.1)	0.731
Current smoker (%)	0 (0)	3 (4.2)	9 (8.5)	2 (5.9)	0.460
Lowest level of education^b^ (%)	1 (6.3)	9 (13.4)	13 (13.8)	1 (3.1)	0.586
Paid employment (%)	6 (35.3)	14 (19.7)	39 (36.8)	4 (11.8)	0.010
Cynical hostility (range 0–10)	3.1 (1.10)	2.6 (2.5)	1.8 (1.8)	1.10 (2.2)	0.034
Depressive symptoms range (0–36)	11.7 (9.5)	8.5 (7.5)	6.3 (5.7)	4.7 (5.6)	<0.001
Telomere length	1.014 (0.063)	1.004 (0.072)	0.998 (0.070)	0.982 (0.075)	0.114
Telomere length+all covariates^c^	1.009 (0.065)	1.003 (0.072)	0.996 (0.070)	0.981 (0.076)	0.201

a Data presented as means (SD) or frequencies (%).

b No qualification.

c Age, BMI, smoking status, education attainment, current employment status, cynical hostility and depressive symptoms.

Average relative telomere length (T/S ratio) of the sample was 0.994 (SD = 0.075). Telomere length was unrelated to gender in this sample. Telomere length was shorter among men who smoked (P = 0.013) and those who had greater hostility scores (P<0.001), but was unrelated to age, BMI, employment status, educational attainment and depression scores in either men or women.

There was a linear association between telomere length and sleep duration in men (P = 0.035) (see Table 2), so that men with greater sleep duration had longer telomeres, independently of age, educational attainment, employment status, BMI, smoking, hostility and depressive symptoms. There was no association between sleep duration and telomere length in women. When sensitivity analyses were performed to remove participants who might have been depressed (using a conventional cut off score of 16) the linear association between sleep duration and telomere length remained statistically significant in men (P = 0.008), and was still not significant among female participants (P = 0.409).

## Discussion

We found that in men sleep hours were related to telomere length in a linear fashion, so men sleeping 5 or fewer hours had the shortest telomere length while those reporting more than 7 hours the longest. These associations were independent of relevant covariates including depressive symptoms.

Evidence relating sleep with telomere length in population-based studies is limited. Apart from studies of sleep apnea [20], [21] the relationship between sleep measures and telomere length has been explored only in women [17], [18]. Prather et al. [18] found no association between telomere length and sleep duration in a study of 245 middle-aged women, although sleep quality was inversely related with telomere length. This relationship was independent of age, race, income, BMI and perceived stress. A second study of over 4000 women from the Nurses' Health Study revealed that short sleep duration (≤6 hours) was related to shorter telomere length only in participants younger than 50 years, and not in older women [17]. In our study women were aged 64 years on average, so this may be why we did not see an association in women. The linear association between sleep duration and telomere length in men has not been reported before, to the best of our knowledge.

The mechanisms through which sleep might be related to telomere attrition are yet to be established. Although sleep duration was unrelated to gender in our analyses, the literature suggests that men experience poorer sleep as they grow older [28] and tend to have shorter telomere length than women as well as more rapid telomere attrition [4], [9]. Short sleep duration and telomere length are both associated with inflammation [4], [11], [29]. Oxidative stress may also be implicated, since prolonged sleep deprivation leads to increased oxidative stress [30] and is associated with exacerbated telomere shortening [31]. Short sleep duration and telomere length are also both associated with increased sympathetic tone, since shorter sleep duration increases sympathetic nervous system activity [32], [33]. Sleep duration may impact telomere length through neuroendocrine pathways as well since stress and a dysregulated diurnal rhythm of stress mediators, such as flat slope or high evening levels of cortisol, are associated with shorter sleep duration [34], [35]. Stress, conditions of chronic adversity and increased cortisol levels have also been associated with shorter telomere length [6], [36]. *In vitro*, Choi et al. [37] has demonstrated that exposure to high levels of cortisol is associated with a reduction of telomerase activity, the main enzyme involved in telomere maintenance.

Dysfunctional telomeres are risk factors for adverse health conditions, and may accelerate the progression of age-related disorders as well [38]. In addition to a strong hereditary component, telomere length can be influenced by psychological and sociodemographic characteristics [8], [11], [25]. Modifiable risk factors such as smoking and obesity have also been associated with telomere shortening [9], [10]. Such data are valuable in helping to identify populations who might be at an increased risk for telomere attrition, and consequently subject to more accelerated aging processes. Sleep is another factor that is subject to modification, so could provide an opportunity to ameliorate the health profile at older ages. Our study and Liang et al.'s [17] data, if confirmed by future studies, could offer a new avenue for intervention in aging.

The data are cross-sectional so causal conclusions cannot be drawn as to whether short sleep duration contributes towards telomere shortening, or whether telomere shortening is a marker of biological processes that impair sleep. Although we controlled for age, BMI, smoking education attainment, paid employment, hostility and depressive symptoms, other unmeasured factors could be responsible. Pre-existing illness is unlikely to be the explanation, since respondents in this study were drawn from the Whitehall II study, a well characterized epidemiological cohort that has been studied for more than 20 years, so we are confident that physical and mental illness are not responsible. Sleep was measured by self-report, and this might be affected by current mood and memory biases [39]. An objective measure of sleep duration, such as accelerometry would have been more desirable, but is often prohibited in epidemiological cohorts by financial constraints and additional participant burden. Associations between self-reported and objectively measured sleep duration have been reported previously [40]. Participants were white civil servants and the findings cannot be extrapolated to other populations.

In conclusion, our study adds to the growing literature relating lifestyle factors with telomere length, and we report for the first time that short sleep duration in healthy older men is associated with shorter telomere length. Longitudinal studies are needed to establish whether shorter sleep leads to accelerated telomere shortening and advanced cellular aging.
